# Shaping Efficiency: Parametric Design for Schwedler Domes

**DOI:** 10.3390/ma19091772

**Published:** 2026-04-27

**Authors:** Ahmed Fathy Aly Omar Ibrahim, Katarzyna Jeleniewicz, Artur Piekarczuk

**Affiliations:** 1 Institute of Civil Engineering, Warsaw University of Life Sciences, Nowoursynowska 159, 02-776 Warsaw, Poland; ahmeddfathi2@gmail.com; 2 Building Research Institute, Filtrowa 1, 00-611 Warsaw, Poland; a.piekarczuk@itb.pl

**Keywords:** parametric modeling, structural analysis, Schwedler domes, lattice structures, structural efficiency, lightweight structures, design automation, computational design, interoperability

## Abstract

**Highlights:**

- Up to 31% reduction in structural mass was achieved through the use of closed-section profiles.
- Hybrid configuration (IPE ribs + SHS rings) provided the most efficient structural performance among the three configurations considered, reaching utilization ratios of 0.87 (ribs) and 0.63 (rings).
- Parametric modeling enabled the rapid generation and comparison of multiple design variants within a unified computational workflow.
- A dedicated Python component generates the cladding polylines from the same nodal data used by the structural model so that non-uniform snow loading can be applied per panel group without manual selection in the finite-element environment.
- Parametric workflows enable efficient identification of structurally optimized configurations in lattice dome systems.
- Design automation significantly reduces modeling time while ensuring consistency between geometric and analytical models.
- The proposed approach provides a foundation for integrating structural analysis with automated design and future optimization processes and for connecting the same parametric source to BIM and fabrication environments and to life-cycle assessment.

**Abstract:**

Lightweight structures such as Schwedler domes offer high strength-to-weight ratios for large-span applications; however, their design typically involves time-consuming iterative processes. This study proposes an integrated parametric workflow combining geometry generation, structural analysis, and automated load application to improve both design efficiency and structural performance. The methodology is based on Python scripting within Grasshopper, enabling parametric control of dome geometry and direct interoperability with Autodesk Robot Structural Analysis Professional. Three open-apex Schwedler dome configurations were analyzed as a focused demonstration of the workflow, differing in cross-sectional typology and structural layout. The results show that the use of closed sections reduces structural mass by up to 31%, while hybrid configurations achieve significantly improved member utilization, reaching 0.87 for ribs and 0.63 for rings. Importantly, the parametric workflow enabled the rapid generation and evaluation of multiple design variants, significantly reducing modeling time and eliminating inconsistencies between geometric and analytical models. The study demonstrates that parametric modeling provides an effective framework for designing efficient dome structures, enabling both material optimization and accelerated design processes. The same parametric source is also suitable for extension into BIM and fabrication environments, as well as into life-cycle assessment, which are identified as planned continuations of this research.

## 1. Introduction

Domes have long been recognized as one of the most efficient structural forms, offering high strength-to-weight ratios and the ability to span large areas with minimal material consumption. The steel-framed dome, in particular, has been extensively studied for its structural efficiency and practical applications in large-span construction [1]. Among various dome typologies, Schwedler domes are distinguished by their orthogonal arrangement of meridional ribs and circumferential rings, complemented by diagonal bracing. This structural configuration enables efficient load redistribution and favorable stiffness-to-weight characteristics, as demonstrated in comparative studies of reticulated dome systems [2,3]. In particular, it has been shown that the structural efficiency of Schwedler domes is strongly influenced by geometric parameters such as subdivision density and rise-to-span ratios [3,4,5]. In recent decades, the development of computational design methods has significantly influenced the design of complex spatial structures. Parametric modeling enables the definition of relationships among geometric variables, enabling the systematic exploration of design alternatives. This approach is especially relevant for dome structures, where relatively small changes in geometry may significantly affect internal forces, stiffness, and stability [6,7]. Previous studies have demonstrated that parametric modeling can effectively support conceptual structural design and facilitate performance-based exploration of design variants [6,8].

Parametric and algorithmic design approaches, widely discussed in the literature, enable geometry to be defined through relationships and rules rather than fixed entities, allowing automatic updates of the entire structural model in response to changes in input parameters [9]. Such approaches shift the design process from the direct modeling of geometry to the formulation of generative algorithms, which can be directly linked to structural analysis and optimization procedures [10].

In parametric structural design, two implementation paradigms have emerged. In the first, the finite-element solver is embedded directly in the parametric environment. Karamba3D [11] provides native Grasshopper components for trusses, frames and shells, and more recent tools such as Alpaca4D, used for instance by Sardone et al. [12] in the optimization of geodesic domes, follow the same pattern. The advantage is tight coupling between geometry and analysis. However, the limitation, in the engineering-office context, is that code-compliant member designs and Eurocode verification still require a second platform. In the second paradigm, the parametric environment is used as a geometry and data generator that is live-linked to an industry-standard design software such as Autodesk Robot Structural Analysis through an interoperability layer such as GeometryGym. The present work follows this second paradigm.

A separate research stream, developed in particular by the Block Research Group at ETH Zurich, approaches curved-structure design from a form-finding perspective. Thrust Network Analysis and its implementations, from RhinoVAULT [13] to the broader body of shell form-finding collected in Adriaenssens et al. [14], derive geometry from equilibrium rather than from a prescribed topology. In common engineering practice, lattice steel domes of the Schwedler type are instead specified by their topology (number of meridians, number of parallels, rise-to-span ratio) under architectural and code-compliance constraints. The workflow proposed here adopts that starting point and focuses on automating everything that follows from it.

The applicability of Grasshopper-based parametric modeling to complex curved geometries is not restricted to form-finding or to steel. Recent work on digital stereotomy, such as the PoliBrick plug-in developed by Pourfouladi et al. [15], shows how Python-backed parametric components can produce systematic panelizations of doubly curved masonry shells and expose them as clean data trees ready for structural analysis. The structural physics of a brick vault is distant from that of a steel Schwedler dome, but the pattern is relevant to the present study, which provides a dedicated Python component that concentrates the non-trivial geometric reasoning in one place and avoids a long chain of native Grasshopper logic, which may cause complications in achieving goals such as defining the cladding panel vertices.

At the same time, Building Information Modeling (BIM) has become a standard in the architecture, engineering, and construction industry, enabling the creation of data-rich digital models that support design, fabrication, and construction processes [16,17]. Modern BIM tools, such as Tekla Structures, allow detailed modeling of structural elements, including geometry, materials, and fabrication data, which is particularly important for complex spatial structures [18].

Despite these advances, integrating parametric modeling, BIM environments, and structural analysis tools remains a significant challenge. Differences in data structures, loss of information during data exchange, and limited interoperability between software platforms often lead to inconsistencies between geometric and analytical models [19,20,21,22]. These issues are especially critical in the case of spatial structures, where large numbers of interconnected elements require consistent definition of geometry, boundary conditions, and structural properties [19].

At the same time, structural optimization has become an important research area in civil engineering. Optimization approaches for spatial structures include size, shape, and topology optimization, often supported by advanced computational techniques [23]. In the context of dome structures, optimization studies have confirmed that structural performance depends on a complex interaction between geometry, topology, and cross-sectional properties and that efficient solutions require integrated consideration of these aspects [24].

Despite the significant body of research, several important limitations can be identified. First, many studies focus either on parametric form generation or on structural optimization without providing a unified computational workflow that directly links geometric modeling with structural analysis and design verification and with onward BIM and fabrication environments. Second, parametric studies are often limited to geometric exploration, with insufficient consideration of engineering aspects such as member grouping, cross-sectional differentiation, and realistic load transfer mechanisms, including the automated application of spatially differentiated snow loading, as specified in EN 1991-1-3 [25]. Third, interoperability among parametric, BIM, and analytical environments remains unresolved, limiting the practical applicability of these approaches in structural engineering design [19,20,21].

Another important aspect concerns the analytical modeling of dome behavior. It has been demonstrated that lattice domes are highly sensitive to geometric nonlinearity and initial imperfections, particularly in lightweight configurations [26]. In particular, low-rise dome structures should be analyzed using second-order (geometrically nonlinear) formulations, as linear analysis may underestimate displacement and stability effects [27].

Furthermore, the specific case of open-apex Schwedler domes remains relatively underexplored. The presence of a central opening affects stiffness distribution, internal force paths, and global stability, yet this configuration has not been widely investigated within parametric and automated design frameworks.

These limitations indicate a clear research gap: there is a lack of integrated parametric workflows for Schwedler domes that combine automated geometry generation, direct interoperability with structural analysis tools, realistic load modeling, and engineering-oriented design verification within a single computational environment and that remain extensible from the same Python-authored source to BIM and fabrication environments.

Accordingly, the present study proposes a parametric workflow for the design and structural assessment of open-apex Schwedler domes, integrating Python-based modeling in Grasshopper with structural analysis in Autodesk Robot Structural Analysis Professional. The contribution consists, firstly, of a Python-authored geometry component that builds the nodes and the rib, ring, and brace segments of a trimmed (open-apex) Schwedler dome and outputs the orientation data required by the downstream GeometryGym components. Secondly, a dedicated Python-authored cladding component produces the panel polylines from the same nodal data so that subsequent panel partitioning into uniform and non-uniform snow-loading groups is performed on a clean, script-generated dataset. This enables us to make a live link to Autodesk Robot Structural Analysis and get automated geometry that includes the claddings, section assignment, support and release definition, and different load-case applications. Also, the same geometry Python-authored source can serve as a basis for planned extensions toward BIM and fabrication environments (Tekla Structures) and toward life-cycle assessment, explicitly identified here as continuing work.

The study investigates the influence of the number of meridians and the cross-sectional typology on structural efficiency, with particular emphasis on member utilization and structural weight. Geometrically nonlinear analysis and initial imperfections are incorporated to ensure the realistic representation of structural behavior. The three configurations analyzed in this paper are a focused demonstration of the workflow, and they probe two specific design dimensions, which are the cross-sectional typology and the rib count. The number of possible models can vary depending on the target or design needs.

In this way, the study contributes both a focused evaluation of design strategies for Schwedler domes and a reproducible computational workflow that bridges parametric modeling, BIM-based design, and structural engineering practice.

## 2. Materials and Methods

This section describes the computational framework developed for the parametric design, structural analysis, and optimization of open-apex Schwedler domes. The methodology integrates parametric geometric modeling, algorithmic scripting, and finite-element analysis (FEA) within a unified parametric environment to enable efficient design iteration and automated evaluation of structural performance. A dome with a diameter of 20 m, a height of 10 m, and eight rings was adopted as the reference geometry for all analyzed models, with variations introduced in cross-sectional profiles and the number of ribs (meridians). A complete workflow diagram of the parametric workflow is given in Figures S3 and S4, and the two Python components driving it are specified at the level of algorithmic pseudocode in Figures S1 and S2 (see Supplementary Materials).

### 2.1. Software and Tools

The development of the parametric framework relied on a coherent set of computational tools and plug-ins operating within a Windows-based environment. The selected components ensured interoperability and seamless data exchange between geometric modeling, parametric control, and structural analysis modules, which are essential for integrated computational design workflows in structural engineering.

#### 2.1.1. Geometric Modeling and Parametric Environment

Rhinoceros 3D (Version 8) (Robert McNeel & Associates, Seattle, WA, USA) served as the primary geometric modeling platform, providing the geometric foundation for all subsequent operations. Grasshopper 1.0.0008, a visual programming plug-in integrated within Rhino 8, provided the interface for developing the parametric algorithms and workflows. Standard Grasshopper components were employed for data management, flow control, and the real-time visualization of the intermediate and final geometric configurations.

#### 2.1.2. Core Logic and Scripting

Core geometric generation logic was implemented using Python scripting via the Python 3.9.10 script component available within Grasshopper. This approach was selected for its flexibility, scalability, and computational efficiency, particularly for handling iterative and condition-based operations that are difficult to implement using native visual components alone. The script utilized the Rhino Geometry library to programmatically construct and manipulate geometric entities. The Python code was partially developed with the assistance of large language models (Claude 3.5 Sonnet, Anthropic, San Francisco, CA, USA; Gemini 1.5 pro, Google, Mountain View, CA, USA), using both traditional and iterative vibe coding approaches. These tools were employed to accelerate code writing, explore alternative algorithmic strategies, and assist in debugging. All generated code was critically reviewed, validated, and tested by the authors to ensure correctness, reproducibility, and compliance with the research objectives.

#### 2.1.3. Structural Analysis Interoperability

The seamless transfer of geometric models to the structural analysis software was achieved through the GeometryGym 24.10.28.8 plug-in [28]. This plug-in provides a suite of Grasshopper components designed to create analysis models compatible with various structural analysis platforms. In this workflow, ggRhinoRobot components were used to generate models directly compatible with Autodesk Robot Structural Analysis (ARSAP) (Version 2025) (Autodesk, San Francisco, CA, USA), which served as a finite-element analysis (FEA) platform.

### 2.2. Parametric Geometry Generation

The parametric engine, developed within Grasshopper using Python scripting, constitutes the core component of the proposed computational framework. This module is responsible for generating the geometric definition of a trimmed Schwedler dome based on a set of user-defined input parameters.

#### 2.2.1. Input Parameters

The Python script receives input parameters through a set of Grasshopper controls (number sliders or panels), enabling real-time modification of the dome geometry and immediate propagation of changes throughout the computational workflow, as shown in Table 1.

The change made to these input parameters triggers an automatic recalculation in the Python script, updating all downstream geometry and analysis definitions.

#### 2.2.2. Geometric Generation Logic

**Figure 1:**
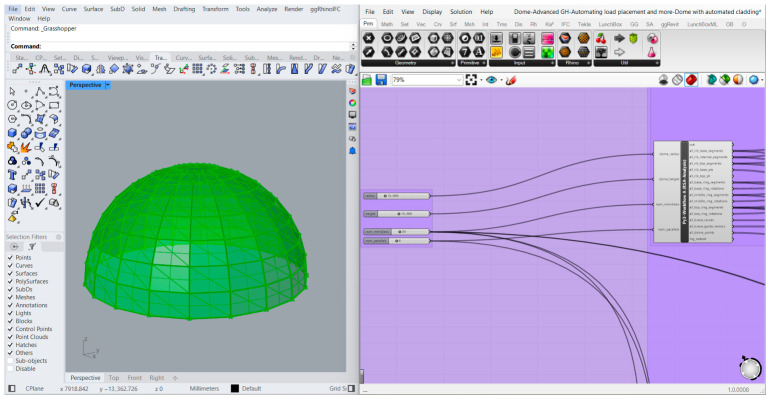
Grasshopper interface demonstrating parametric generation of a trimmed Schwedler dome geometry via input parameters (**right side**) and the resulting geometric output previewed in Rhino (**left side**).

The Python script employs the fundamental principles of spherical geometry to calculate the node coordinates of the Schwedler dome, and this results in the generation of the dome geometry, as shown in Figure 1. The complete generative logic is also stated at the level of language-agnostic pseudocode as Algorithm 1, as shown in Figure S1 (see Supplementary Materials).

The script begins by validating all inputs to ensure they are of the correct type and within reasonable bounds (e.g., radius > 0, num meridians ≥ 3). Default values are applied when inputs are missing, and warning or error messages are logged for invalid inputs to assist with debugging. Based on the input radius R and height H, the script determines the properties of the governing sphere segment. The maximum polar angle $\theta_{max}$ measured from the vertical axis is defined as

(1) $$\theta_{max}=\cos^{-1}\left(\frac{R-H}{R}\right)$$

For each parallel level j, the vertical angle ψ is calculated as

(2) $$\psi =\theta_{max}\left(1-\frac{j}{n_{parallels}}\right)$$

The corresponding vertical coordinate is determined as

(3) $$Z=\left(H-R\right)+R\cos\left(\psi \right)$$

The corresponding horizontal radial distance is determined as

(4) $$r_{xy}=R\sin\left(\psi \right)$$

The script identifies the highest level for which $r_{xy}>0$, defining the upper boundary of the structural system and enabling the creation of a trimmed (open-apex) dome configuration.

For each meridian i, the circumferential angle ϕ is computed as

(5) $$\phi =\frac{2\pi i}{n_{meridians}}$$

The Cartesian coordinates of each node are then obtained using

(6) $$X=r_{xy}\cos\left(\phi \right)\;Y=r_{xy}\sin\left(\phi \right)$$

The computed coordinates are stored as Rhino.Geometry.Point3d objects and organized into structured datasets representing nodal topology.

The script subsequently generates structural elements by systematically connecting nodes according to the Schwedler dome topology:

- ribs (vertical elements);
- rings (horizontal elements);
- diagonal bracing members.

Rib elements are generated as discrete linear segments between adjacent levels, ensuring compatibility with finite-element modeling requirements. These elements are categorized into base, intermediate, and top segments, enabling the independent assignment of boundary conditions and release definitions. Ring elements are created at each parallel level by connecting adjacent nodes along the same horizontal plane. These elements are similarly categorized based on their position (base, middle, top). Diagonal bracing members are generated by connecting nodes between adjacent levels, forming triangulated substructures that enhance global stiffness and stability.

To ensure the proper definition of local coordinate systems in the structural analysis model, the script calculates orientation parameters for each element. For ring elements, rotation angles are derived from the inclination of the corresponding rib elements. For diagonal braces, auxiliary guide vectors are computed to maintain consistent local axis orientation relative to the dome surface.

**Figure 2:**
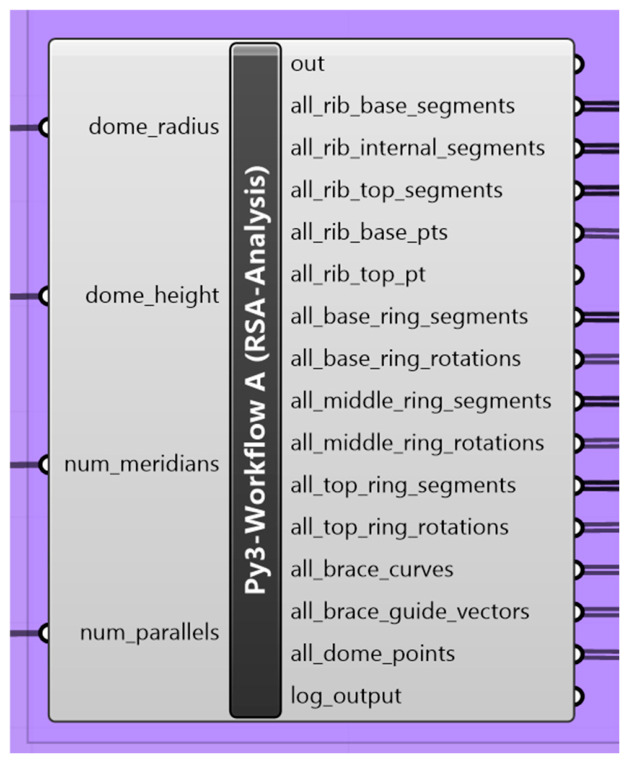
Screenshot for the geometry Python component showing the inputs and outputs.

Finally, the script produces multiple structured outputs, including geometry, connectivity, and orientation data, which are subsequently used for automated structural model generation and design optimization procedures, as shown in Figure 2.

### 2.3. Analysis Model Generation

The workflow for generating a structural analysis model directly compatible with Autodesk Robot Structural Analysis was developed using ggRhinoRobot, the Geometry Gym plug-in inside Grasshopper. This process translates the parametrically generated geometry into a fully defined finite-element model.

#### 2.3.1. Segmentation Strategy

A critical consideration in the workflow is the handling of curved members (ribs and rings). Since FEA software such as ARSAP represents structures using nodes connected by linear elements, curved members must be discretized appropriately to ensure accurate connectivity and structural behavior.

To address this, the parametric model generates individual linear segments between adjacent nodes rather than continuous curves. This approach ensures the correct interpretation of the geometry, connectivity, and stiffness distribution within the finite-element framework.

Furthermore, rib elements are subdivided into base, intermediate, and top segments, enabling the independent assignment of boundary conditions and release definitions. This segmentation allows for the precise control of structural behavior, such as assigning fixed or pinned conditions at selected locations.

#### 2.3.2. Parametric Model Definition in ARSAP

**Figure 3:**
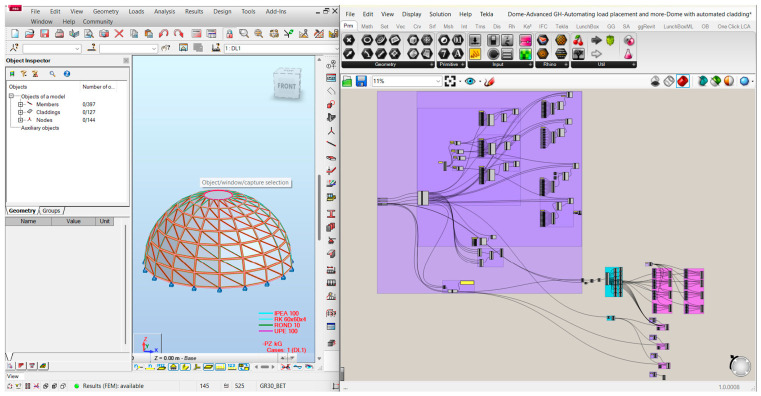
Screenshot from GH and ARSAP showing the overall script on the right and the generated model in ARSAP on the left. Background colors indicate different script functions. Cyan represents cladding logic, lavender magenta indicates load application, and periwinkle covers all other geometric details.

The outputs from the Python script were fed into various ggRhinoRobot components to build the ARSAP model parametrically and to be generated as shown in Figure 3.

The structural model was generated using ggRhinoRobot components, which allow parametric definition of all the essential elements of the analysis model.

Material properties were defined using the ggRobotCreateElasticIsotropicMaterial component, where structural steel (e.g., S235) was assigned. Cross-sectional properties were defined using ggRobotCreateSectionProp components, with profiles assigned separately for ribs, rings, and bracing members.

Structural members were created using the ggRobotCreateBar component, which assigns both geometry and section properties to each element. The orientation of elements was controlled using parametrically computed rotation angles and guide vectors, ensuring consistent local axis definition across the structure.

Boundary conditions were defined using ggRobotSupport components, allowing full parametric control over translational and rotational constraints. Similarly, member end releases were defined using ggRobotBarRelease components, enabling the explicit control of degrees of freedom at element ends, which is essential for the realistic modeling of connections in lattice structures.

#### 2.3.3. Automated Model Assembly and Data Consistency

The integration of parametric geometry with analysis components enables the fully automated generation of the structural model, including the geometry, materials, cross sections, supports, releases, and load definitions.

This approach eliminates manual model reconstruction in the FEA environment and ensures data consistency between geometric and analytical representations, significantly reducing the risk of modeling errors.

Additionally, the workflow supports the rapid regeneration of the model following any modification of input parameters, enabling the efficient exploration of multiple design configurations within a consistent analytical framework.

### 2.4. Automated Cladding Generation

Generation of the dome cladding followed a three-stage geometric translation process. This ensured that the abstract mathematical points of the dome were converted into discrete structural entities compatible with ARSAP for load application and analysis.

#### 2.4.1. Stage I: Data Structuring (Partitioning)

The initial geometric output from the parametric model consisted of a flat list of nodal coordinates representing the dome vertices. To enable panel generation, this data was reorganized using structured data trees, in which each branch corresponds to a horizontal ring (parallel) and each element within the branch corresponds to a specific meridian position.

This transformation enables systematic access to the nodal relationships required for panel definition and ensures topological consistency across the dome surface.

#### 2.4.2. Stage II: Quadrilateral Panel Generation

A dedicated Python script was used to generate quadrilateral cladding panels by iteratively connecting adjacent nodes between consecutive parallel levels. For each pair of neighboring rings, panels were defined using four vertices forming closed polylines, ensuring compatibility with surface element generation in the analysis model. The corresponding pseudocode is given as Algorithm 2 in Figure S2 (see Supplementary Materials).

To account for the circular topology of the dome, periodic boundary conditions were implemented using modular indexing, ensuring seamless closure of the geometry without discontinuities along the meridional direction.

The algorithm was designed to exclude the apex region, resulting in a trimmed (open-apex) dome configuration. The upper boundary was instead defined by a separate top ring, which serves as the termination of the structural and cladding system.

Additionally, the panel generation procedure allows the segmentation of cladding into multiple independent subsets, enabling differentiated load application. In the present study, the dome surface was divided into multiple panel groups corresponding to different elevation zones and circumferential regions, which is particularly useful for modeling non-uniform snow load distributions. A close-up view is presented below in Section 2.5.2.

#### 2.4.3. Stage III: Structural Integration

The generated cladding panels were subsequently converted into finite-element surface elements within ARSAP, allowing automated load distribution and interaction with the structural model. This integration enables direct coupling between geometric panelization and structural analysis, ensuring that load application is consistent with the actual discretized geometry of the dome.

### 2.5. Load Definition and Application

#### 2.5.1. Load Calculation Basis

**Figure 4:**
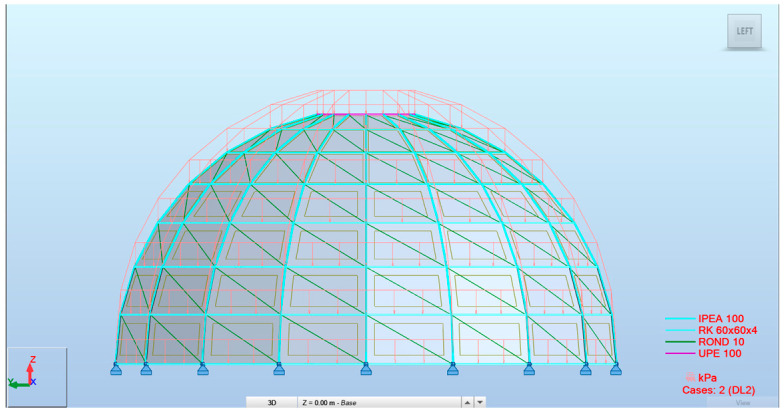
DL2 placement in ARSAP.

Load values were calculated externally in accordance with relevant Eurocodes (EN 1990, EN 1991-1-1, EN 1991-1-3, and EN 1991-1-4) [25,29,30,31] and then incorporated into the parametric workflow. In the current implementation, the per-case load magnitudes are computed outside the parametric environment and supplied as numerical vector components. All loads were applied automatically to the generated cladding panels within the finite-element model, ensuring consistency across all analyzed configurations. Five load types were considered in the analysis: self-weight of the structural members (automatically calculated in ARSAP) (DL), additional dead load (cladding and installations) (DL2), imposed (live) load (LL), uniform snow load (SN1), non-uniform (drifted) snow load (SN2), and wind load (WL). DL2 was applied as a uniform load to all cladding panels, as shown in Figure 4. The values of the DL2 and LL were calculated as follows: DL2 = 0.34 kN/m^2^, LL = 0.4 kN/m^2^.

**Figure 5:**
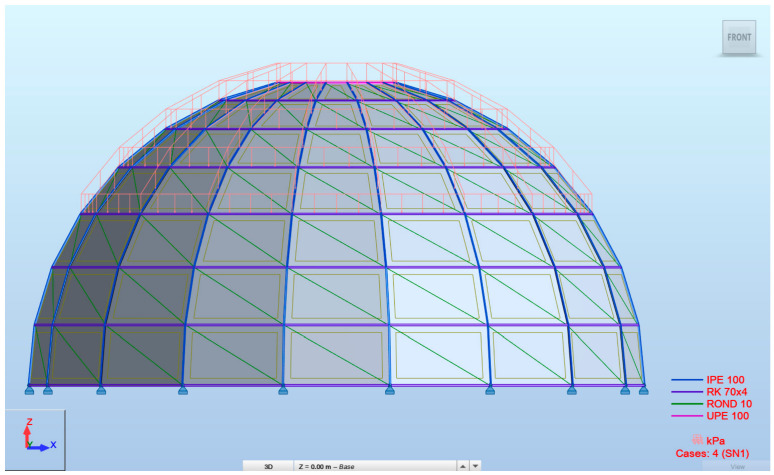
Snow load case (SN1) placement in ARSAP.

Snow loads were calculated according to EN 1991-1-3 [25], assuming a location in Tarczyn, Poland (snow load zone II), at an elevation of 140 m above sea level. The uniformly distributed snow load was applied as shown in Figure 5 and equaled $0.8\;kN/m^{2}$.

**Figure 6:**
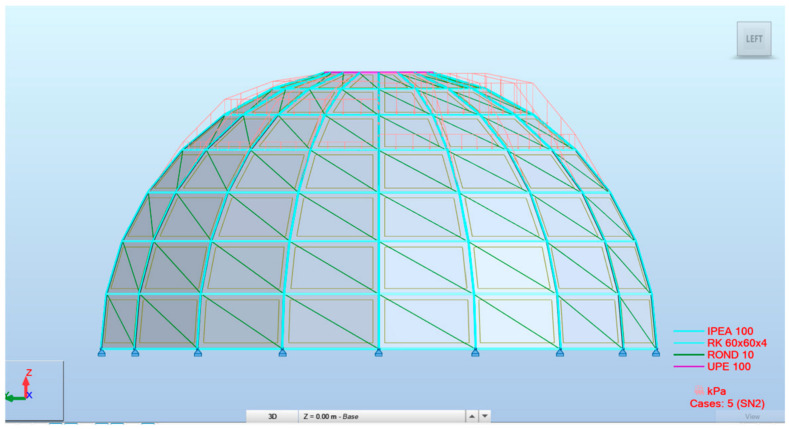
Snow load case (SN2) placement in ARSAP.

The non-uniformly distributed snow load was calculated as 1.8 kN/m^2^ and 0.9 kN/m^2^ and placed as shown in Figure 6.

Wind load was generated using the built-in wind simulation module in ARSAP based on the dome geometry and a reference wind velocity of 22 m/s. The load was automatically distributed over the structure according to the software’s implementation of Eurocode-based wind actions.

#### 2.5.2. Load Application and Automation

**Figure 7:**
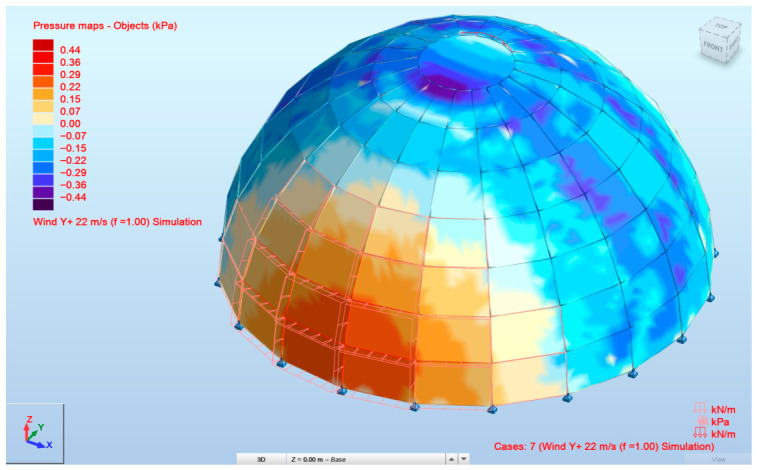
Wind load placement in ARSAP.

The wind load is shown in Figure 7.

**Figure 8:**
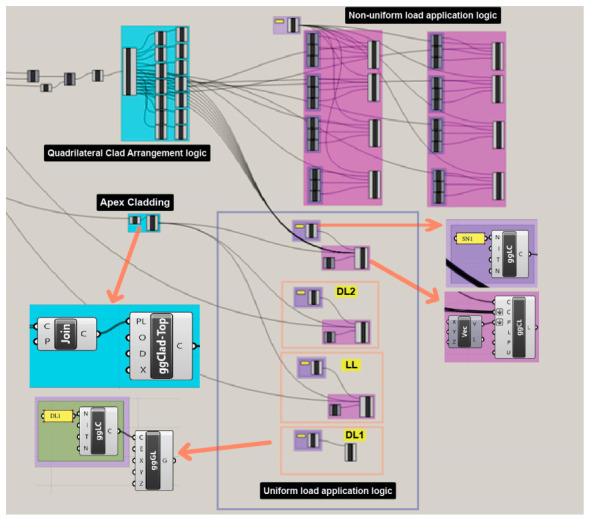
Wide view from GH Script showing the logic of forming the cladding panels and applying different load cases.

The load application was fully integrated within the Grasshopper environment, enabling the automated assignment of load cases to parametrically generated cladding panels. The overall logic of the cladding formation and load distribution is illustrated in wide view in Figure 8.

**Figure 9:**
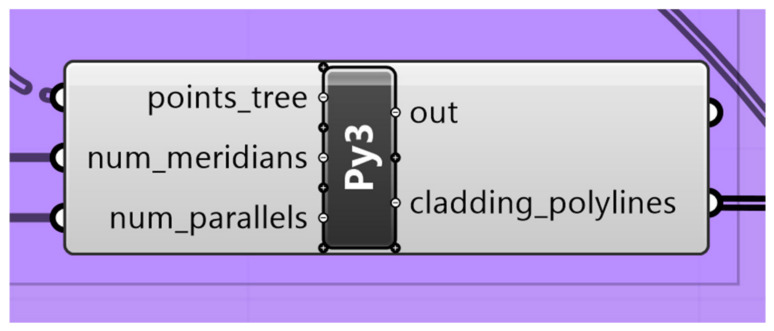
The Python component that generates the cladding polylines.

To manage the complex geometry of the dome, the process begins with the identification and organization of the panel boundaries. As shown in Figure 9, the Python component is utilized to extract the cladding polylines of the main geometry.

**Figure 10:**
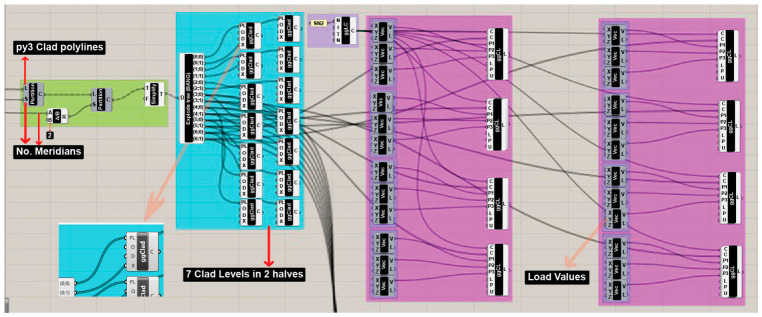
The cladding arrangement logic alongside the non-uniform load application.

Once these polylines are identified, they are organized into specific data branches to facilitate targeted load assignments. As shown in Figure 10, a series of partitioning and simplifying components are used to divide the cladding into seven distinct levels, with each level further split into two halves to accommodate non-uniform requirements.

The script then moves into the specific logic for load cases. For uniform distributions like dead and live loads, the top apex cladding and the main output of the quadrilateral cladding polylines formed by Python scripting are joined. Non-uniform snow loads require a more granular approach. The previously organized data branches allow for precise targeting. The final mapping ensures that each panel group receives its designated load input accurately within the global model. This approach ensures that load definitions remain consistent with geometric modifications, allowing rapid and reliable evaluation of multiple design variants within a unified computational framework. By establishing these rules within Python and Grasshopper, we can generate any number of dome models while maintaining the same accuracy for structural load assignment.

### 2.6. Computational Workflow and Analysis Assumptions

All the components described above were integrated into a single parametric workflow implemented within the Grasshopper environment, enabling seamless interaction between geometric modeling, structural analysis, and load definition. The complete pipeline is summarized in Figures S3 and S4 (see Supplementary Materials) within four stages.

The input parameters, including the geometric variables (radius, height, number of meridians, and number of parallels), material properties, and cross-sectional definitions, were controlled using parametric input components (e.g., number sliders and panels). These inputs were directly linked to the Python-based geometry generation module, ensuring fully automated propagation of parameter changes throughout the entire workflow.

Any modification of input parameters triggered automatic regeneration of the geometric model, structural analysis model, and load definitions, eliminating the need for manual updates and ensuring consistency between all stages of the process. Beyond the three configurations analyzed in this study, the workflow responds predictably to any parameter changes. A change in any of the four geometry sliders (radius, height, number of meridians, number of parallels) propagates through both Python components and regenerates every downstream object—the nodes, ribs, rings, brace segments, cladding polylines, and the panel subsets feeding the load cases. A change in a cross-sectional profile or material grade updates only the corresponding ggSP or ggEIM component outputs, while the geometry remains unchanged. A change in a single load magnitude, whether uniform or non-uniform, updates only the corresponding ggLoad component, while the rest of the model is unchanged. In every case, the rebuild inside Grasshopper is operated by the component graph without manual intervention. Re-sending the model through the live link and re-running the analysis inside ARSAP remains a user-initiated step. This behavior makes the workflow reproducible for any open-apex Schwedler dome configurations within the bounds of the input validation performed by the Python scripts.

The developed workflow was used to generate three distinct dome configurations for comparative analysis, each consisting of eight rings and differing in the number of ribs and assigned cross-sectional profiles. These three configurations were selected as a focused demonstration of the workflow. However, the Python-authored geometry component can generate any number of open-apex dome variants by changing the input sliders. The three instances analyzed here were chosen to probe cross-sectional typology (open vs. closed section) and meridian count (20 vs. 18 ribs).

The analyzed configurations included

- a 20-rib reference model with IPE profiles assigned to both ribs and rings;
- a 20-rib model with SHS profiles for both ribs and rings;
- an 18-rib model with hybrid IPE (ribs) and SHS (rings) profiles.

This sequence of models was intentionally defined to progressively evaluate the influence of cross-sectional typology on structural performance, starting from uniform open-section solutions, through closed-section systems, to hybrid configurations tailored to the structural role of individual members.

All models were analyzed using structural steel grade S235, with Young’s modulus E=210 GPa.

Load combinations were generated automatically within ARSAP in accordance with EN 1990 (PN-EN 1990:2004/AC:2010) [32].

Both first-order (linear) and second-order (geometrically nonlinear) analyses were performed. However, the evaluation of structural performance and the conclusions presented in this study are based primarily on second-order analysis results due to the sensitivity of lattice dome structures to geometric nonlinearity and stability effects.

In addition, initial geometric imperfections were introduced in the rib elements to account for local instability phenomena and to ensure realistic representation of structural behavior. The imperfections were applied in accordance with standard engineering practice, providing a conservative assessment of the load-bearing capacity of the structure.

For the purpose of structural design and verification, the dome members were classified into four groups: vertical elements (ribs), horizontal elements (rings), bracing members, and the upper compression ring. This grouping enabled independent cross-sectional optimization and more accurate assessment of structural performance for each category of elements.

The design (member sizing and verification) was carried out using Autodesk Robot Structural Analysis Professional (ARSAP), in accordance with relevant Eurocode provisions. The utilization ratios were obtained directly from the software based on the defined load combinations and structural analysis results.

## 3. Results

**Figure 11:**
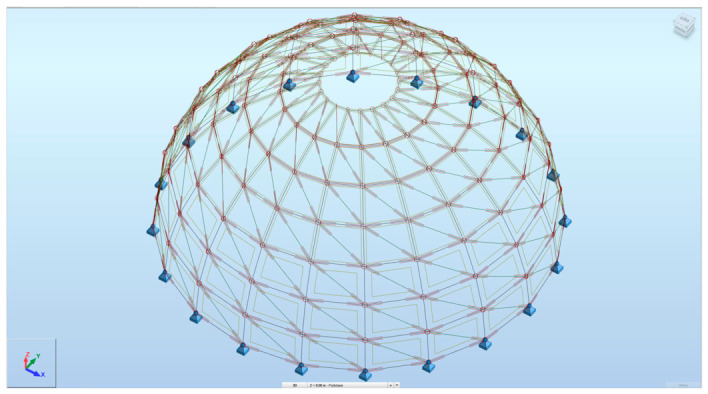
The resulting dome model in ARSAP after generating it through Grasshopper.

**Figure 12:**
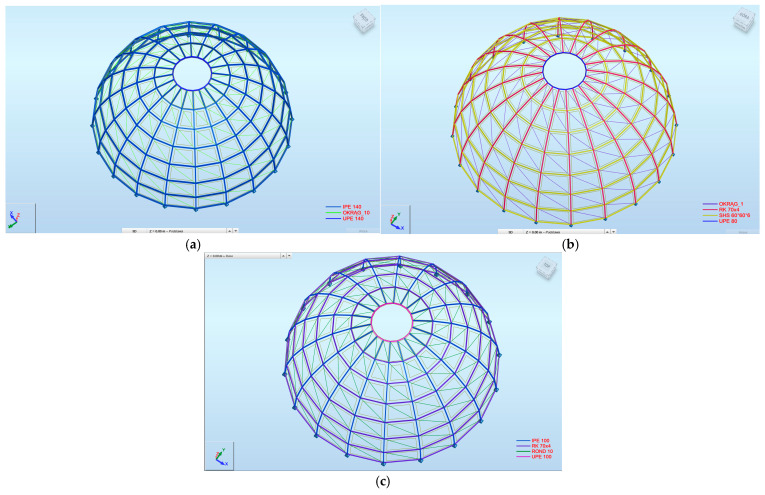
Parametrically generated Schwedler dome models in ARSAP: (**a**) Model 1; (**b**) Model 2; (**c**) Model 3.

The developed parametric workflow enabled the fully automated generation of structural models in Autodesk Robot Structural Analysis Professional directly from the Grasshopper environment (Figure 11 and Figure 12). All analyzed configurations were derived from a single parametric definition, ensuring consistency of geometry, boundary conditions, and load application across all variants. This approach allowed the rapid generation and direct comparison of multiple design alternatives without manual model reconstruction. The three configurations reported in this section probe two specific design dimensions of the parametric model, cross-sectional typology and rib count, while the remaining dimensions of the design space are held at the reference values (20 m diameter, 10 m height, eight rings, structural steel S235). The conclusions drawn from the comparison are accordingly scoped to this setting.

The three representative structural configurations analyzed are as follows: a 20-rib dome with IPE sections (Model 1), a 20-rib dome with SHS sections (Model 2), and an 18-rib dome with a hybrid configuration consisting of IPE ribs and SHS rings (Model 3).

The structural performance of the analyzed configurations is summarized in Table 2. A significant variation in total structural mass was observed between the models. Model 1 exhibited the highest mass of 3540 kg, indicating inefficient material usage associated with a uniform open-section design. In contrast, Model 2 achieved the lowest mass of 2450 kg, corresponding to a 31% reduction compared to Model 1. Model 3 resulted in a mass of 2684 kg, representing a 24% reduction relative to Model 1.

**Figure 13:**
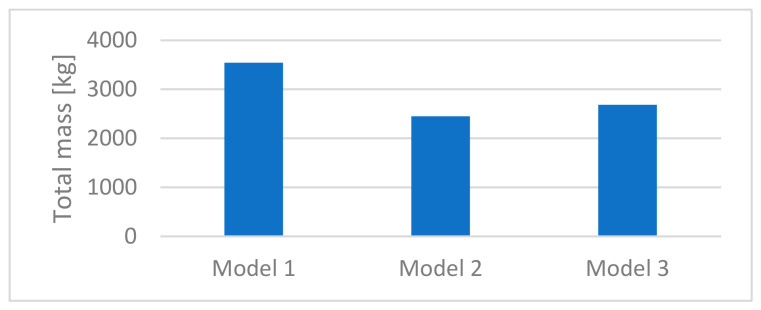
Comparison of total structural mass for the analyzed dome configurations.

Although Model 2 provides the lowest structural mass (Figure 13), its utilization levels remain relatively low, indicating the underutilization of structural capacity. This confirms that minimizing structural weight alone does not guarantee optimal structural performance.

A detailed analysis of utilization ratios reveals significant differences between structural configurations and member groups. In Model 1, utilization remained low (0.33 for ribs and 0.70 for rings), indicating overdesigned members. Model 2 showed similar utilization for ribs (0.33) and reduced utilization for rings (0.43), suggesting inefficient load distribution despite its reduced weight.

In contrast, Model 3 achieved the most balanced and efficient structural response, with rib utilization reaching 0.87 and ring utilization 0.63, indicating near-optimal use of material capacity. This behavior indicates that the design of Model 2 was governed by serviceability limit state (SLS) requirements, particularly displacement constraints, rather than ultimate limit state (ULS) conditions. Consequently, the structural members remained underutilized in terms of strength capacity despite achieving a reduced overall mass. These results demonstrate that hybrid cross-sectional strategies enable better alignment between internal forces and the structural resistance of individual members.

The influence of structural layout is particularly evident in Model 3, where the reduction in the number of ribs from 20 to 18 resulted in increased load demand in circumferential members and the improved global utilization of structural elements. Within the parametric framework, this modification required only a change of a single input parameter, which automatically updated the entire structural model.

**Figure 14:**
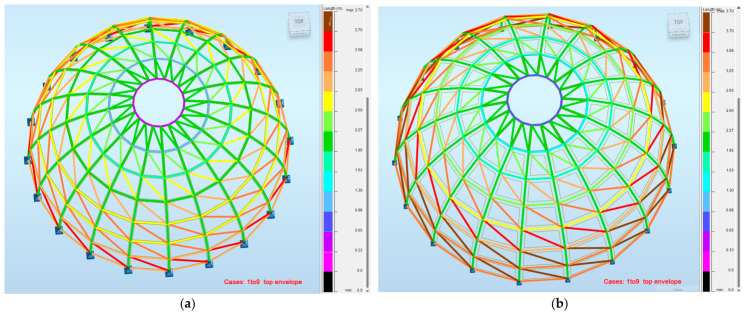
Distribution of structural member lengths for ribs and rings in the analyzed Schwedler dome configurations: (**a**) Model 2; (**b**) Model 3.

Figure 14 illustrates the distribution of structural member lengths for the analyzed configurations. The results show that the reduction in the number of ribs leads to an increase in the length of individual members, particularly in the meridional direction. This has a direct influence on structural behavior, as longer members are more sensitive to buckling and require more efficient cross-sectional design.

From a parametric design perspective, this confirms that geometric parameters, such as the number of ribs, not only affect structural topology but also significantly influence member slenderness and, consequently, the overall structural performance.

To better understand the differences in structural performance between the analyzed configurations, a mechanical interpretation based on internal force distribution and stiffness characteristics was performed.

**Figure 15:**
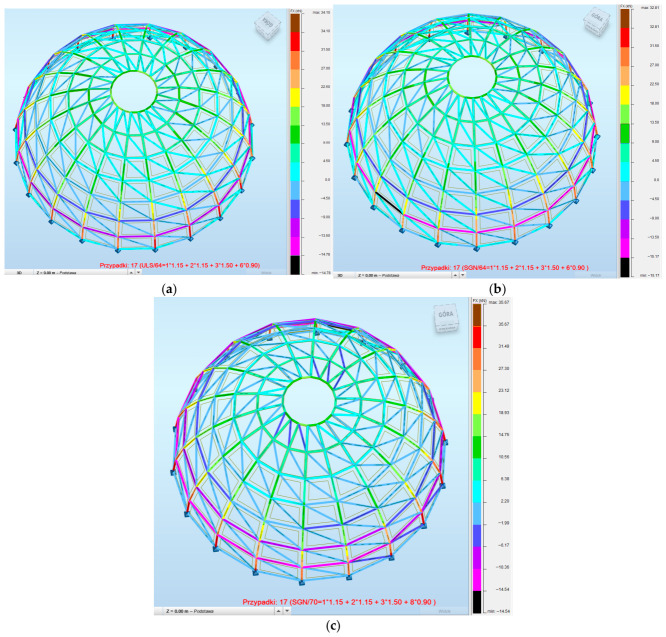
Distribution of axial forces in ribs and rings: (**a**) Model 1; (**b**) Model 2; (**c**) Model 3.

A comparative analysis of internal force distribution across the three configurations reveals distinct differences in structural behavior (Figure 15). Model 1 is characterized by highly uneven force distribution, with only a limited number of members actively participating in load transfer. Model 2 exhibits a more uniform distribution; however, the overall force levels remain low due to increased structural stiffness, resulting in the underutilization of capacity. In contrast, Model 3 demonstrates a well-balanced structural response, with efficient load redistribution and higher engagement of primary structural members. This confirms that structural efficiency is governed by the interaction between geometry, stiffness, and cross-sectional configuration rather than by weight reduction alone.

**Figure 16:**
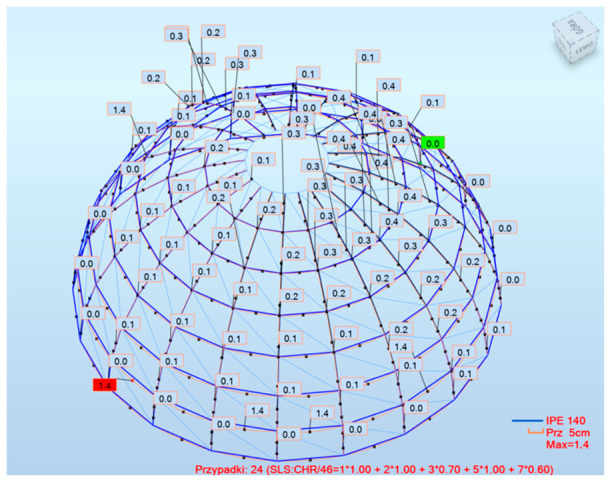
Model 1—Deflection of dome structures under governing load combinations.

The maximum deflection remained within a comparable range for all models, with values of 14 mm, 12 mm, and 15 mm for Models 1, 2, and 3, respectively. Despite increased utilization and reduced structural redundancy, Model 3 did not exhibit excessive deformation, confirming that improved material efficiency does not compromise global stiffness. Figure 16 presents an example of displacements in the dome structures under the governing load combinations.

**Figure 17:**
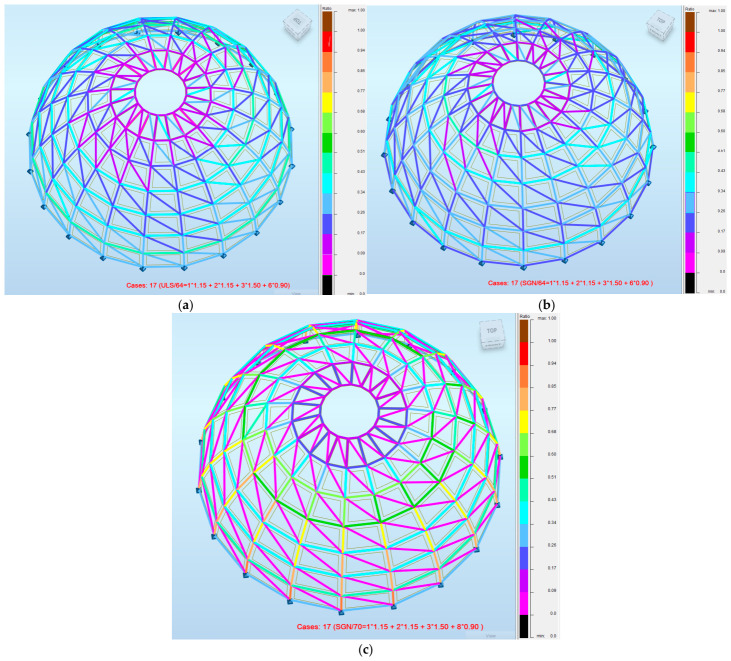
Distribution of utilization ratios in analyzed dome configurations: (**a**) Model 1; (**b**) Model 2; (**c**) Model 3.

Finally, the distribution of utilization across the structures confirms the advantages of a hybrid design. Model 1 shows significant areas of underutilization, while Model 2 exhibits more uniform but still suboptimal behavior. Model 3 demonstrates a more efficient stress distribution, with higher utilization concentrated in primary load-carrying members. Figure 17 presents the distribution of utilization ratios for the analyzed dome configurations, providing insight into the efficiency of load transfer within the structural system.

In Model 1, large areas of low utilization can be observed, indicating significant overdesign and inefficient material distribution. The structural response is characterized by the uneven engagement of members, with only selected elements contributing effectively to load transfer.

Model 2 exhibits a more uniform distribution of utilization; however, the overall levels remain relatively low. This confirms that, despite the reduction in structural mass, the system does not fully utilize its load-bearing capacity, which is consistent with the governing serviceability limit state.

In contrast, Model 3 demonstrates a more efficient and targeted distribution of internal forces. Higher utilization values are concentrated in primary load-carrying members, while secondary elements remain within safe limits.

This indicates that the hybrid configuration enables better alignment between structural demand and member capacity, resulting in a more efficient structural system.

From a parametric design perspective, these results highlight the capability of the developed workflow to capture and visualize the redistribution of internal forces resulting from changes in geometry and cross-sectional typology, supporting informed design decisions.

Overall, the results indicate that uniform cross-section solutions lead to inefficient material usage, while closed sections improve weight efficiency but do not fully utilize structural capacity. The hybrid configuration provides the best balance between weight reduction and structural performance.

Importantly, the parametric modeling framework enabled the rapid generation, modification, and evaluation of these design variants, demonstrating its effectiveness as a tool for the efficient structural design of dome systems.

## 4. Discussion

The results confirm that parametric modeling provides an effective framework for improving both structural efficiency and design workflow in the analysis of Schwedler domes.

The comparison of the analyzed configurations shows that cross-sectional typology has a significant influence on structural performance. The use of SHS sections in Model 2 resulted in the lowest structural mass; however, the relatively low utilization levels indicate that the design was governed by serviceability limit state (SLS) requirements rather than ultimate limit state (ULS) conditions. This confirms that minimizing structural weight alone does not guarantee optimal structural efficiency. A similar observation has been reported in studies on lattice domes, where lightweight configurations are often governed by displacement and stability criteria rather than strength [33,34].

In contrast, the hybrid configuration (Model 3) achieved significantly higher utilization levels, particularly in rib elements, while maintaining acceptable displacement values. This demonstrates that structural members should be designed according to their specific load-carrying roles rather than using uniform cross sections. This finding is consistent with optimization-based studies of dome structures, which emphasize the importance of adapting cross-sectional properties to internal force distribution [17,23]. The present study focuses on comparative evaluation of structural performance within a parametric framework. A detailed stability assessment, including buckling modes and ultimate load-carrying capacity, was not included and is considered an important direction for future research.

The influence of structural layout is also evident. The reduction in the number of ribs resulted in increased load redistribution and improved utilization of structural elements. This indicates that the controlled reduction of structural redundancy can enhance material efficiency when combined with appropriate cross-sectional selection. Similar conclusions have been reported in comparative studies of reticulated domes, where structural efficiency was shown to depend strongly on geometric configuration and member arrangement [3].

The parametric workflow proposed here can be compared with existing approaches in structural parametric design in three ways. With respect to parametric-native finite-element plug-ins such as Karamba3D [11] and Alpaca4D [12], the present workflow relies on a live link to an industry-standard Eurocode design software rather than on a solver embedded in Grasshopper; this choice trades the tightness of coupling for access to established code-compliance procedures that remain the basis of engineering-office design in many jurisdictions. With respect to form-finding methods such as Thrust Network Analysis and its implementations [13,14], the proposed workflow starts from a prescribed Schwedler topology, as is usual in the practical design of such domes, and focuses on automating the downstream stages rather than deriving the form itself from equilibrium. With respect to Python-backed parametric tools for curved structures such as PoliBrick [15], the present cladding component follows the same organizing idea, which is to concentrate non-trivial geometric reasoning in one script and expose clean data to the downstream pipeline applied to a different structural system. The workflow is therefore complementary to these approaches.

A key contribution of this study is the integration of parametric modeling with structural analysis. The developed workflow enables the automatic generation of geometry, structural models, and load definitions, significantly reducing modeling time and eliminating inconsistencies between geometric and analytical models. This addresses a well-documented challenge in the literature related to interoperability between parametric design tools and structural analysis environments [19,20,21].

The ability to automatically generate cladding and apply load cases, including non-uniform snow loads, demonstrates that parametric workflows can support complex engineering tasks while maintaining computational efficiency. Previous studies have highlighted the potential of parametric modeling in conceptual design stages [6,8,35], while the present work extends this capability toward detailed structural analysis and design verification.

The current level of automation is demonstrated by several distinct processes. The generation of the nodal geometry and the various structural segments for the ribs, rings, braces, and top rings is managed by the Python geometry component. Similarly, the Python cladding component handles the creation of all cladding panels. Material assignments, section properties, supports, and member releases are driven by the GeometryGym components. The assignment of load cases to the specific panel groups, including the spatially differentiated non-uniform snow case, is operated entirely inside Grasshopper. Finally, the complete model is transferred to Autodesk Robot through the live link to be analyzed.

Despite these advantages, the study has several limitations. The analysis was conducted for a single geometric configuration, and the results may vary for different dome geometries or loading conditions. Furthermore, the investigated models represent selected design strategies rather than the results of a fully automated optimization process.

Future research will address these limitations through several developments. A primary direction involves performing load calculations parametrically and connecting them directly to the geometry parameters, storing the code equations and design guidelines within the parametric environment itself. This would eliminate the current manual step of external load calculation and enable fully automated load-case generation based on geographic location and geometric configuration. Another capability to be developed is linking the same parametric geometry to building information modeling software for fabrication automation, enabling seamless transfer from optimized design to digital fabrication and construction and life-cycle assessment from the same parametric source. The script will also be made more advanced to parametrically control the remaining design and analysis steps, moving toward a fully automated workflow where all aspects of structural design, from conceptual geometry to code-compliance checking, are integrated within the parametric environment.

The significance of this work lies in demonstrating that parametric design can be fully integrated with structural analysis to produce optimized, code-compliant structural designs, positioning computational design as a bridge between architectural geometry and structural engineering. As sustainability imperatives intensify and digital workflows become standard, approaches that reduce material consumption while accelerating design processes will become essential. The present study represents a step toward that future, a demonstration of current possibilities and a foundation for increasingly automated and intelligent design systems that can contribute to a more efficient and sustainable built environment.

## 5. Conclusions

This study demonstrates that parametric modeling provides an effective and practical framework for the design and analysis of Schwedler domes, enabling both improved structural efficiency and a significant reduction in modeling effort.

The conducted analysis of three representative dome configurations leads to the following key conclusions:

- The type of cross-sectional profile has a decisive influence on structural performance. The use of closed sections (SHS) resulted in a 31% reduction in structural mass compared to open-section solutions; however, this did not lead to the optimal utilization of structural capacity.
- Structural efficiency cannot be evaluated based solely on weight reduction. The lowest-mass configuration (Model 2) exhibited low utilization levels due to governing serviceability limit state (SLS) conditions, indicating underutilization of material capacity.
- Hybrid cross-sectional configurations provide the most efficient structural solution. The combination of IPE ribs and SHS rings enabled near-optimal utilization levels (0.87 for ribs and 0.63 for rings) while maintaining acceptable displacement values.
- Reduction in structural redundancy improves material efficiency. Decreasing the number of ribs from 20 to 18 resulted in more effective load redistribution and higher utilization of primary structural members.
- Geometric parameters directly influence structural behavior. Changes in the number of ribs affect member lengths, slenderness, and load transfer mechanisms, which must be considered in the design process.

Most importantly, the study confirms that parametric modeling enables the rapid generation, modification, and evaluation of multiple design variants within a unified computational environment. The integration of geometry generation, structural analysis, and load application significantly reduces modeling time and eliminates inconsistencies between geometric and analytical models.

The developed workflow demonstrates that parametric design can serve as an effective decision-support tool in structural engineering, allowing engineers to identify optimal configurations more efficiently than with conventional modeling approaches.

The proposed methodology provides a foundation for further development toward fully automated structural design, including integration with optimization algorithms and BIM-based fabrication processes.

## Figures and Tables

**Table 1:** Input parameters of the parametric Schwedler dome model.

Parameter	Type	Description	Constraints
Dome radius (R)	Float	Radius of the dome’s base circle (governing sphere)	>0
Dome height (H)	Float	Overall height of the dome from base to apex level	≥0
Number of meridians	Integer	Number of main radial ribs	≥3
Number of parallels	Integer	Number of horizontal ring divisions along the height (excluding the base)	≥1

**Table 2:** Comparison of structural mass and key performance indicators for the analyzed dome configurations.

Model	No. of Ribs	Cross Sections	Total Mass [kg]	Max Utilization [-]—Vertical/Horizontal Profile	Max Deflection [mm]
Model 1	20	IPE/IPE	3540	0.33/0.70	14
Model 2	20	SHS/SHS	2450	0.33/0.43	12
Model 3	18	IPE/SHS	2684	0.87/0.63	15

## Data Availability

The data presented in this study are available on request from the corresponding author due to ongoing research.

## Supplementary Materials

The following supporting information can be downloaded at: https://www.mdpi.com/article/10.3390/ma19091772/s1, Figure S1. Language-agnostic pseudocode of the geometry Python component driving the parametric engine; Figure S2. Language-agnostic pseudocode of the Cladding Python component driving the parametric engine; Figure S3. The parametric workflow (part 1/2); Figure S4. The parametric workflow (part 2/2).

## Abbreviations

The following abbreviations are used in this manuscript:

ARSAP	Autodesk Robot Structural Analysis Professional
GH	Grasshopper
ggSP	ggRobotCreateSectionProp component
ggEIM	ggRobotCreate Elastic Isotropic Material component
