# Supplementary material for: Shaping Efficiency: Parametric Design for Schwedler Domes
Source: Materials (Basel). 2026 Apr 27;19(9):1772. doi: 10.3390/ma19091772 (PMC13164486; doi:10.3390/ma19091772)
Supplement: Supplementary file 1 [file materials-19-01772-s001.zip › materials-4263239-supplementary.pdf]

Algorithm 1. Dome-geometry generation

Language-agnostic description of the geometry Python component driving the parametric engine

INPUT

```
R — dome radius (R > 0)
H — dome height (H ≥ 0)
Nm — number of meridians (Nm ≥ 3)
Np — number of parallels (Np ≥ 1)
```

OUTPUT (14 data outputs + log)

```
all_dome_points — flat list of vertices (Np+1 × Nm)
rib segments: all_rib_base / internal / top _segments
ring segments: all_base / middle / top _ring_segments
+ ring rotations, rib base & top points, braces & guides
```

Step 1. Validate inputs and compute the governing spherical cap.

```
// Defaults are applied if any input is missing or out of range.
if      H = 0           then   θmax ← π / 2
else if    H ≥ 2R       then   θmax ← π
else   θmax ← arccos( (R − H) / R )
```

Step 2. Generate nodal coordinates on the spherical cap.

```
// Sweep the polar angle from base (j = 0) to apex (j = Np) and the meridional angle around the axis.
for   j = 0, 1, …, Np      do
    ψ ← θmax · (1 − j / Np)
    z ← (H − R) + R · cos(ψ)
  rxy ← R · sin(ψ)
  if  rxy > tol   then   jtop ← j    // highest ring with non-zero radius — recorded here, used later for classification
  for   i = 0, 1, …, Nm − 1   do
      φ ← 2π · i / Nm
      V[j, i] ← ( rxy · cos φ, rxy · sin φ, z )
```

Step 3. Build rib segments and classify them by position along the rib.

```
// Segments are generated between consecutive ring levels; the first is the base, the last is the top, the rest are internal.
for   each rib i = 0, 1, …, Nm − 1    do
  S ← [ Line( V[j, i], V[j + 1, i] )    for   j = 0, 1, …, jtop − 1 ]
  if  |S| = 1   then   rib_base.append(S[0])
  else if    |S| ≥ 2    then   rib_base.append(S[0]), rib_top.append(S[−1]), rib_internal.extend(S[1 : −1])
```

Step 4. Build ring segments, classify them by level, and compute the ring rotation angle.

```
// One segment per meridian, modular closure; level 0 → base, level jtop → top, otherwise middle. Rotation is stored on each
segment.
for   j = 0, 1, …, jtop     do  for   i = 0, 1, …, Nm − 1    do
  s ← Line( V[j, i], V[j, (i + 1)    mod    Nm] )
  α ← ( inclination of the rib at node (j, i) + 180° )  mod  360°
  if  j = 0          then    ring_base.append((s, α))
  else if    j = jtop      then    ring_top.append((s, α))
  else   ring_middle.append((s, α))
```

Step 5. Build diagonal braces and their guide vectors.

```
// A brace runs from the lower ring at (j, i + 1) to the upper ring at (j + 1, i). Guide vectors orient the brace section in
space.
for   j = 0, 1, …, jtop − 1     do   for   i = 0, 1, …, Nm − 1    do
  pBR ← V[j, (i + 1)       mod  Nm]    // lower-ring point
  pTL ← V[j + 1, i]                    // upper-ring point
  brace ← Line( pBR, pTL )
  g ← ring-up at pBR, rotated by α around the local ring tangent
  all_brace_curves.append(brace)
  all_brace_guide_vectors.append(g)
```

Step 6. Return.

```
// 14 data outputs plus the log are exposed on the Grasshopper component; each corresponds to a variable in the script.
return ( all_dome_points, all_rib_base_segments, all_rib_internal_segments, all_rib_top_segments,
all_rib_base_pts, all_rib_top_pts, all_base_ring_segments, all_middle_ring_segments, all_top_ring_segments,
all_base_ring_rotations, all_middle_ring_rotations, all_top_ring_rotations, all_brace_curves, all_brace_guide_vectors,
log_output )
```

NOTE

Every vertex is generated for j = 0, …, Np in Step 2, but rib, ring and brace segments are generated only up to jtop — the highest ring level with non-zero radius. This is what leaves the apex open when the top ring would otherwise collapse to a single point.

Figure S1. Language-agnostic pseudocode of the geometry Python component driving the parametric engine

Algorithm 2. Cladding-polyline generation

Language-agnostic description of the cladding Python component driving the parametric engine

INPUT

```
points_tree — the dome vertices arranged as a data tree,
one branch per ring level
Nm — number of meridians (integer)
Np — number of parallels (integer)
```

OUTPUT

```
cladding_polylines — flat list of closed
quadrilateral polylines, ordered by (ring j, meridian i);
returned as the GH component output variable a.
```

Step 1. Initialise and set the tolerance.

```
cladding_polylines ← [ ]
tol ← Rhino.zero.tolerance        // used to reject degenerate edges
```

Step 2. Iterate over consecutive pairs of ring levels.

```
// Stop one ring short of the topmost branch so that each iteration has a well-defined pair (cur, nxt).
for   j = 0, 1, …, Np − 2      do
   cur ← branch( j )          // lower ring (Nm expected points)
   nxt ← branch( j + 1 )      // upper ring (Nm expected points)
```

Step 3. Check the integrity of the two rings before building any quad.

```
// Skip the whole ring pair if either ring is empty or does not have exactly Nm points.
if  cur is empty  or  |cur| ≠ Nm   or  nxt is empty   or  |nxt| ≠ Nm   then
  skip this ring pair and continue with the next j
```

Step 4. Build the four vertices of one quadrilateral panel, using modular closure.

```
// The modular index (i + 1) mod Nm wraps the last meridian back to the first so the ring closes without a seam.
for   i = 0, 1, …, Nm − 1   do
   p0 ← cur[ i ]
   p1 ← cur[ (i + 1)     mod  Nm ]
   p2 ← nxt[ (i + 1)     mod  Nm ]
   p3 ← nxt[ i ]
   quad_points ← [ p0, p1, p2, p3, p0 ]        // ordered vertex list (closing back to p0)
```

Step 5. Reject degenerate quads.

```
// If any of the four edges is shorter than tol , the quad is degenerate and is skipped; no Polyline is constructed.
if  dist(p0, p1) < tol     or  dist(p1, p2) < tol     or  dist(p2, p3) < tol     or  dist(p3, p0) < tol
then   continue with the next i
```

Step 6. Construct the polyline, validate it, and append.

```
// Build the Polyline from the ordered vertex list, check its validity, and only then add it to the output list.
polyline ← Polyline( quad_points )
if  polyline.IsValid    then   cladding_polylines.append(polyline)
```

```
return   cladding_polylines
```

Figure S2. Language-agnostic pseudocode of the Cladding Python component driving the parametric engine

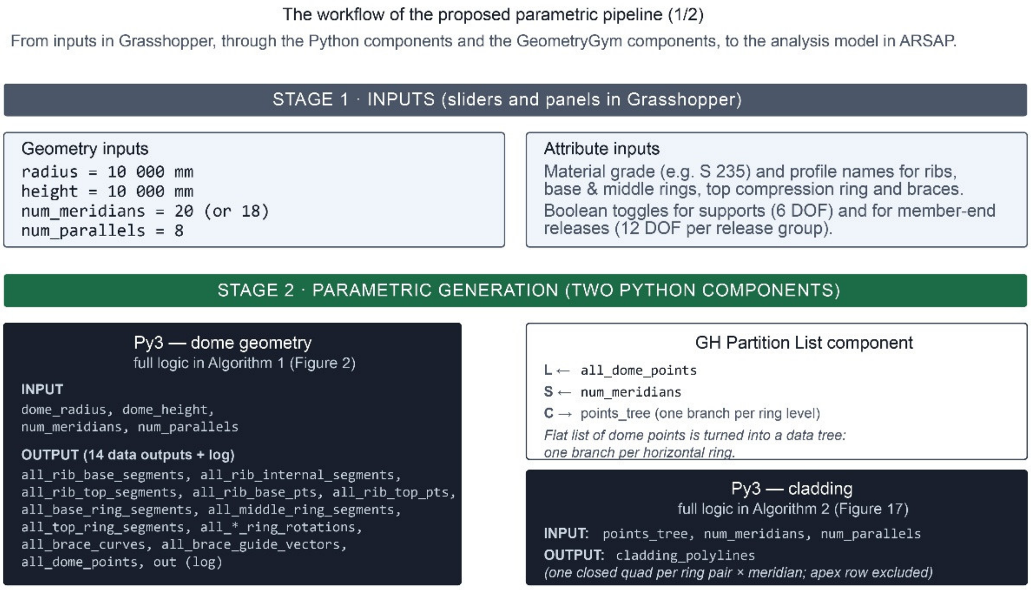

Figure S3. The parametric workflow (part 1/2)

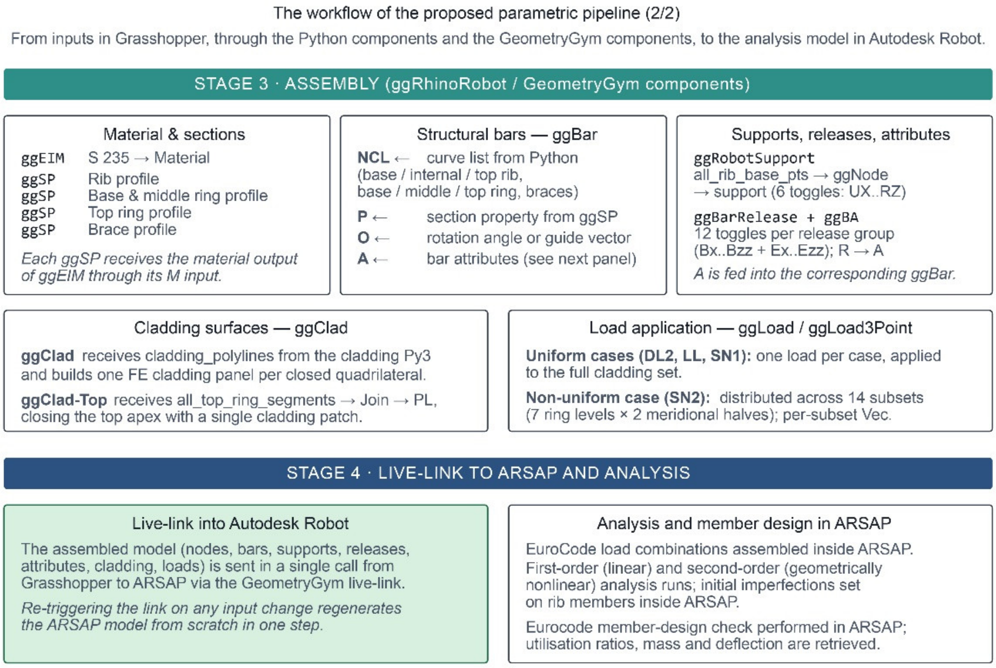

Figure S4. The parametric workflow (part 2/2)
